# Purification and Characterization of Novel Anti-MRSA Peptides Produced by *Brevibacillus* sp. SPR-20

**DOI:** 10.3390/molecules27238452

**Published:** 2022-12-02

**Authors:** Nuttapon Songnaka, Monthon Lertcanawanichakul, Albert M. Hutapea, Sucheewin Krobthong, Yodying Yingchutrakul, Apichart Atipairin

**Affiliations:** 1 School of Pharmacy, Walailak University, Nakhon Si Thammarat 80161, Thailand; 2 School of Allied Health Sciences, Walailak University, Nakhon Si Thammarat 80161, Thailand; 3 Faculty of Science, Universitas Advent Indonesia, Bandung 40559, Indonesia; 4 Center of Excellence in Natural Products Chemistry (CENP), Department of Chemistry, Faculty of Science, Chulalongkorn University, Bangkok 10330, Thailand; 5 National Omics Center, National Science and Technology Development Agency, Pathum Thani 12120, Thailand; 6 Drug and Cosmetics Excellence Center, Walailak University, Nakhon Si Thammarat 80161, Thailand

**Keywords:** antimicrobial peptides, anti-MRSA substances, *Brevibacillus* sp. SPR-20, peptide sequencing, purification

## Abstract

Methicillin-resistant *Staphylococcus aureus* (MRSA) is listed as a high-priority pathogen because its infection is associated with a high mortality rate. It is urgent to search for new agents to treat such an infection. Our previous study isolated a soil bacterium (*Brevibacillus* sp. SPR-20), showing the highest antimicrobial activity against *S. aureus* TISTR 517 and MRSA strains. The present study aimed to purify and characterize anti-MRSA substances produced by SPR-20. The result showed that five active substances (P1–P5) were found, and they were identified by LC-MS/MS that provided the peptide sequences of 14–15 residues. Circular dichroism showed that all peptides contained β-strand and disordered conformations as the major secondary structures. Only P1–P4 adopted more α-helix conformations when incubated with 50 mM SDS. These anti-MRSA peptides could inhibit *S. aureus* and MRSA in concentrations of 2–32 μg/mL. P1 (NH_2_-VVVNVLVKVLPPPVV-COOH) had the highest activity and was identified as a novel antimicrobial peptide (AMP). The stability study revealed that P1 was stable in response to temperature, proteolytic enzymes, surfactant, and pH. The electron micrograph showed that P1 induced bacterial membrane damage when treated at 1× MIC in the first hour of incubation. The killing kinetics of P1 was dependent on concentration and time. Mechanisms of P1 on tested pathogens involved membrane permeability, leakage of genetic material, and cell lysis. The P1 peptide at a concentration up to 32 μg/mL showed hemolysis of less than 10%, supporting its safety for human erythrocytes. This study provides promising anti-MRSA peptides that might be developed for effective antibiotics in the post-antibiotic era.

## 1. Introduction

Antibiotic resistance is a serious problem worldwide. The World Health Organization (WHO) encourages many research sectors to tackle this challenging issue. Methicillin-resistant *Staphylococcus aureus* (MRSA) is one of the most dangerous pathogens. It contains a *mecA* gene that encodes penicillin-binding protein 2a (PBP2a), resulting in a low binding affinity to β-lactam antibiotics [1]. The incidence of community-associated MRSA infection increased from 13% in 2007 to 43% in 2016 [2]. Such infections caused mortality in several diseases such as staphylococcal pneumonia (30–40%) and bacteremia (15–60%) [3]. The search for new antibiotics to treat MRSA is prioritized as highly urgent by the WHO [4]. 

Soil microorganisms are the largest source of antibiotics. *Streptomyces* sp., *Bacillus* sp., and *Brevibacillus* sp. produce many antimicrobial agents, including antimicrobial peptides (AMPs), that effectively combat antibiotic-resistant pathogens [5]. Bacitracin, gramicidin, and polymyxin are the AMPs used in clinical applications nowadays [6]. Most AMPs consist of 10–100 amino acids, have a net charge of +1 to +5 at neutral pH, and have a hydrophobicity between 40% and 50%. AMPs can form an α-helix, β-sheet, random coil, or a combination of α-helix and β-sheet structures to exert their activities [7]. The advantage of AMPs is the multiple actions on the bacterial cells through the disruption of the cell membrane and the interaction with intracellular targets that are difficult to adapt and resist. Hence, the resistance to AMPs is low, and they have gained much attention in antibiotic discovery [8]. 

Our previous study isolated a potential soil bacterium named *Brevibacillus* sp. SPR-20. Its culture supernatant had the highest activity in that study and exhibited an inhibitory spectrum against *S. aureus* TISTR 517 and MRSA strains comparable to vancomycin [9]. However, the active substances of SPR-20 are unknown and require further characterization to identify their components and determine the mechanism of action. This present study aimed to purify the anti-MRSA substances from SPR-20. We determined their antimicrobial activity and investigated the mode of action and toxicity. 

## 2. Results

### 2.1. Purification of Anti-MRSA Substances

**Figure 1:**
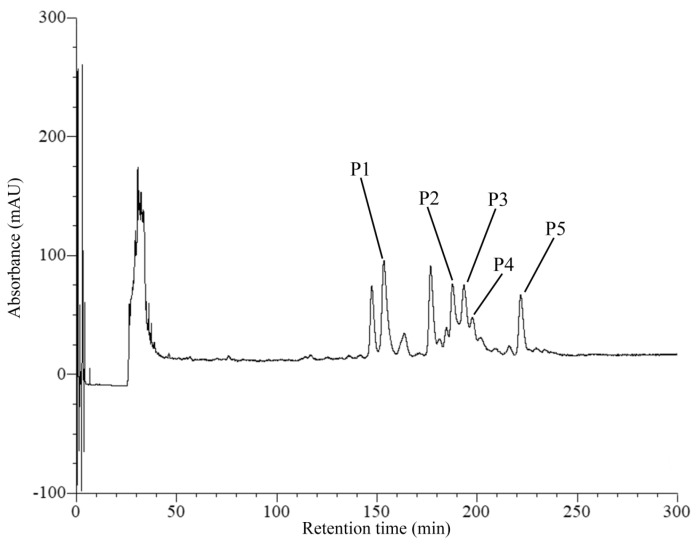
Reversed-phase chromatogram of anti-MRSA substances from SPR-20. The peak signal was monitored at 214 nm. Arrows indicate the active peaks that corresponded to the anti-MRSA activity.

The anti-MRSA substances from the crude supernatant of SPR-20 were purified in three steps. The amount of active substance decreased, and the specific activity increased after each purification step. Ammonium sulfate was added to the supernatant, and the reconstituted precipitate showed an increase in the specific activity (284 AU/mg). The sample was subjected to a cation-exchange column (CIEX), and the specific activity was increased to 452 AU/mg. The reversed-phase column (RPC) was used in the final step, and the result showed that five fractionations exhibited anti-MRSA activity in which P1 was the major component (Figure 1). The specific activity from the P1 fraction was 1694 AU/mg, which corresponded to an 11-fold increase in purity and a yield recovery of 21% (Table 1).

### 2.2. Determination of Antimicrobial Activity by Microdilution Method

The minimum inhibitory concentration (MIC) and minimum bactericidal concentration (MBC) of all antimicrobial substances from SPR-20 were measured. The inhibition of *S. aureus* TISTR 517 and MRSA strains was found in concentrations between 2 and 32 μg/mL. The P1 substance had the highest inhibition with a MIC of 2 μg/mL and was followed by P3 (4 μg/mL), P2/P4 (8 μg/mL), and P5 (32 μg/mL) (Table 2). Vancomycin (2 μg/mL) could inhibit all tested strains. Cefoxitin (2 μg/mL) was only active in response to *S. aureus* TISTR 517, whereas it was insusceptible to MRSA strains as the MIC was more than 64 μg/mL. P1 also exhibited the highest bactericidal activity with MBC of 2 μg/mL and was followed by P3 (4–8 μg/mL), P4 (8–16 μg/mL), P5 (32 μg/mL), and P2 (16–64 μg/mL). In addition, the MBC values of vancomycin and cefoxitin were equal to their MIC values, and the bactericidal effect of cefoxitin on MRSA could not be determined.

### 2.3. Characterization of Anti-MRSA Substances

**Figure 2:**
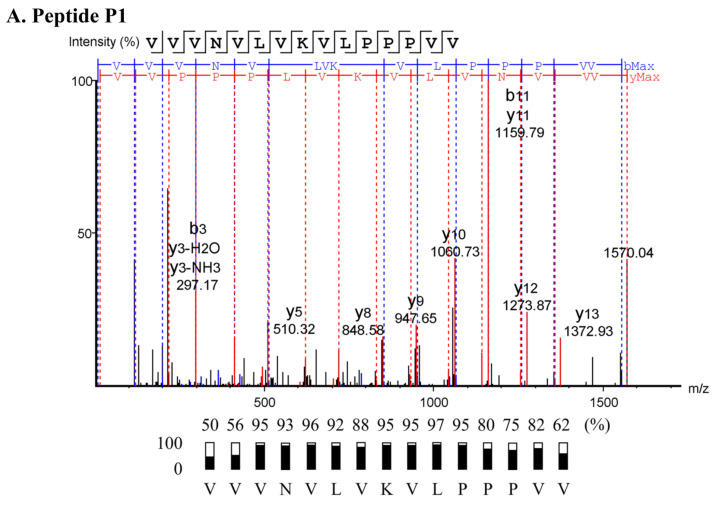

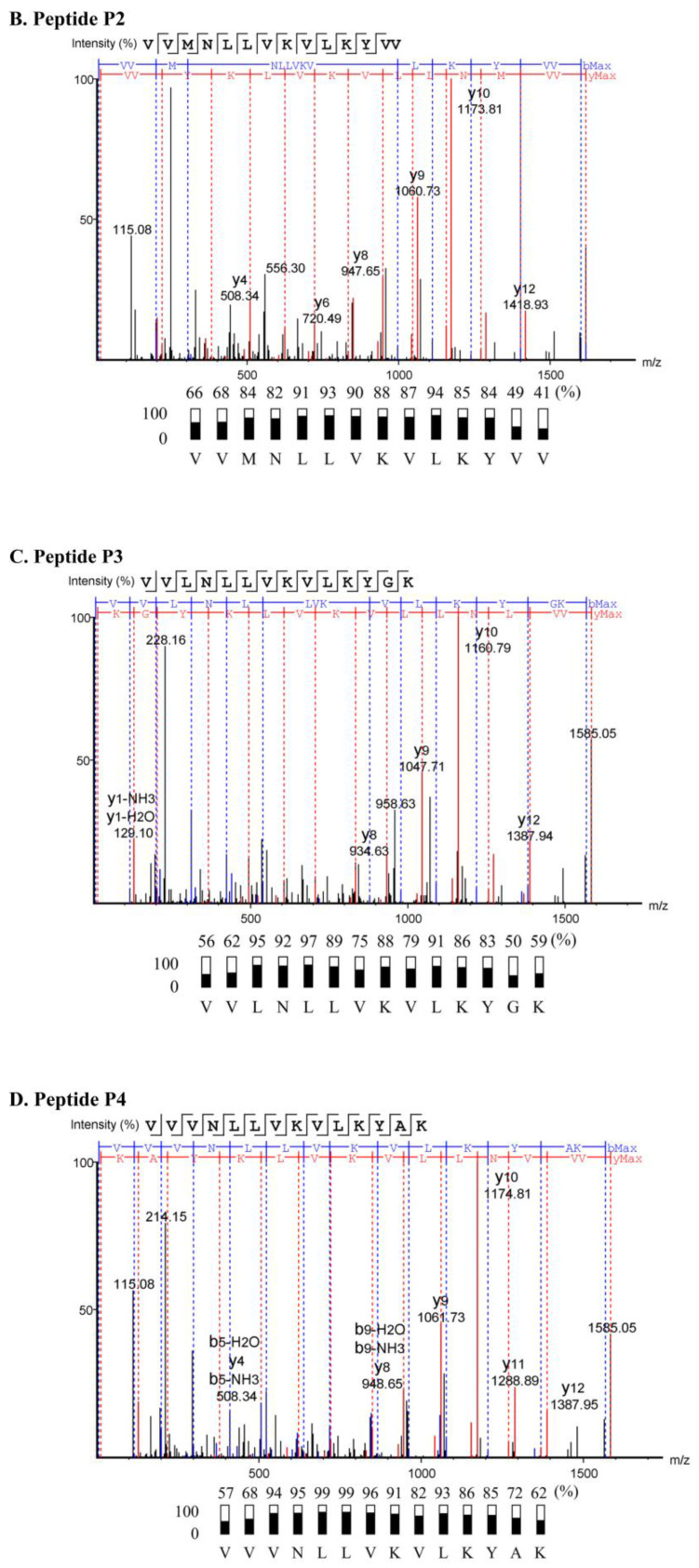

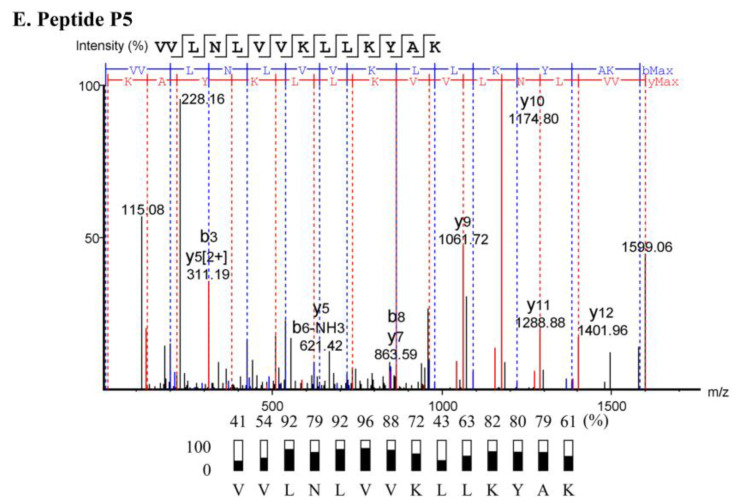
De novo peptide sequencing of the anti-MRSA peptides from SPR-20. The fragmentations to generate the b- and y-ions of five peptides (P1–P5) were analyzed by a tandem mass spectrometer, and the corresponding mass spectra are shown in (**A**–**E**), respectively. The peptide sequences were derived from the mass spectra, and the local confidence scores assigned to each amino acid are also shown.

The active substances were analyzed by liquid chromatography–tandem mass spectrometry (LC-MS/MS). The peptide sequences were fragmented into daughter ions of the b- and y-ion series (Supplementary Material Table S1). A de novo algorithm was used to predict the peptide sequences based on the difference in their mass values for a series of successive peptide ion series (Figure 2). The sequences and their predicted physicochemical properties of five peptides were summarized (Table 3). P2–P5 were composed of 14 residues, and P1 contained 15 amino acids. Their predicted mass ranged from 1570.02 to 1616.01 Da with hydrophobicity between 0.636 and 0.915. The non-polar amino acids, such as valine (V), leucine (L), and proline (P), contributed to these peptide hydrophobicities. The peptides had a theoretical pI between 8.62 and 9.51 and were cationic molecules with the predicted net charges of +1 to +3 at pH 7.4 because of the presence of lysine (K). 

**Figure 3:**
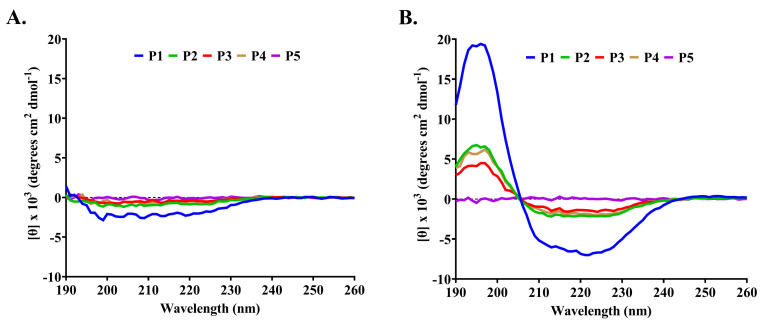
CD spectra of anti-MRSA peptides (P1–P5) from SPR-20. The peptide samples (0.6 mg/mL) were prepared in (**A**) deionized water and (**B**) 50 mM SDS. The CD spectra were measured at 25 °C in the wavelength between 190 and 260 nm.

The secondary structures of P1–P5 were measured by circular dichroism (CD) (Figure 3A). The result showed that all peptides in the water had a negative band between 190 and 230 nm with a great magnitude at around 200 nm that corresponded to the presence of β-strand and random conformations in the structure (>70%) (Table 4). SDS has a negative charge and is used to mimic the anionic environment of the bacterial membrane. The combination of peptides (P1–P4) and SDS resulted in the changes in CD spectra by which a negative band between 205 and 240 nm with a magnitude at 220 nm and a positive band between 190 and 205 nm with a magnitude at 195 nm were observed (Figure 3B). These corresponded to the α-helical characteristic, and the spectral analysis revealed an increase in α-helix conformation when SDS was incorporated into the peptide solutions. P1 apparently showed a spectrum change by which the proportions of the ordered structures for α-helix and β-strand were 4.1% and 44.0% in water and 25.5% and 29.3% in 50 mM SDS, respectively. The structural change of the peptides (P1–P4) in more folded conformations (α-helix and β-strand) was proposed to contribute to the antimicrobial activity upon binding to the negative charge of the cell membrane. In contrast, the CD spectra of P5 in water and SDS were not different, and it was estimated that the percentage contents of α-helix, β-strand, and disordered structures were 3.1, 43.1, and 53.8 in water and 2.6, 40.7, and 56.7 in 50 mM SDS, respectively.

**Figure 4:**
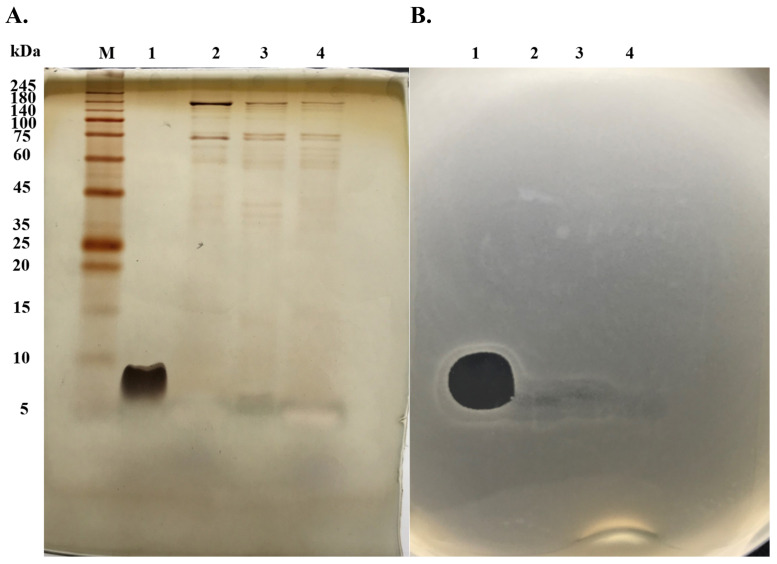
(**A**) Silver-stained SDS-PAGE gel that separated the fractions from each purification step. Lane M, protein marker; Lane 1, P1 from RPC; Lane 2, fraction from CIEX; Lane 3, fraction from ammonium sulfate precipitation; Lane 4, cell-free supernatant. (**B**) Agar overlay assay by which the corresponding gel was overlaid by soft agar containing MRSA isolate 2468. The plate was incubated at 37 °C for 24 h. CIEX, cation exchange chromatography; RPC, reversed-phase chromatography.

The P1 peptide exhibited the highest inhibition of the sensitive and resistant strains of *S. aureus*, so it was used for further evaluation. The result from the SDS-PAGE analysis revealed that P1 appeared as a single band with a molecular weight of less than 10 kDa, whereas the fractions from other purification processes showed impurity bands at a higher molecular weight (Figure 4A). The corresponding gel was overlaid by soft Mueller Hinton (MH) agar containing MRSA isolate 2468, and the inhibition zones were observed at the same position as that of P1, supporting its anti-MRSA activity (Figure 4B). 

### 2.4. Comparison of Potency between Anti-MRSA Peptide and Vancomycin

**Figure 5:**
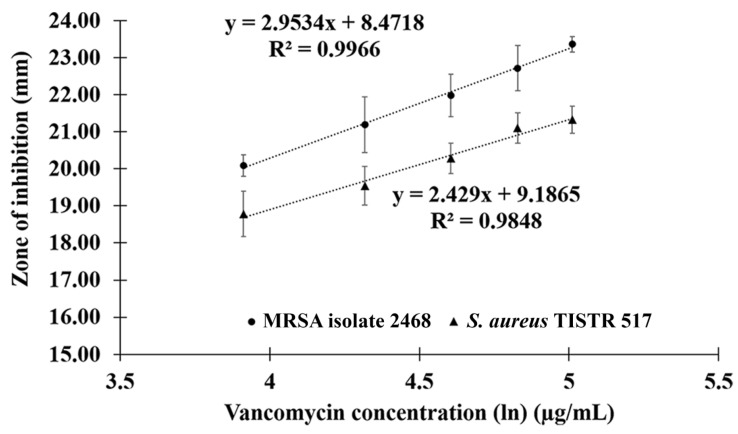
The standard curve for potency comparison between anti-MRSA peptide (P1) and vancomycin. The curve was constructed by plotting between vancomycin concentrations and zone of inhibition against *S. aureus* TISTR 517 and MRSA isolate 2468.

The activity of P1 was evaluated for the equivalent potency compared to vancomycin from the standard curve (R^2^ > 0.98) (Figure 5). Based on the size of the inhibition zone, P1 (15 µg/mL) showed similar antimicrobial activity to vancomycin at the concentrations of 75–100 μg/mL and 100 μg/mL against *S. aureus* TISTR 517 and MRSA isolate 2468, respectively (*p*-value > 0.05). The potency of P1 (15 µg/mL) was equivalent to that of vancomycin at 76.29–87.36 and 85.97–109.05 µg/mL for *S. aureus* TISTR 517 and MRSA isolate 2468, respectively (Table 5). This indicated that P1 was about 5.09–5.82- and 5.73–7.27-fold more potent than vancomycin against those bacteria, respectively.

### 2.5. Scanning Electron Microscopy (SEM) of Anti-MRSA Peptide-Treated Pathogens

**Figure 6:**
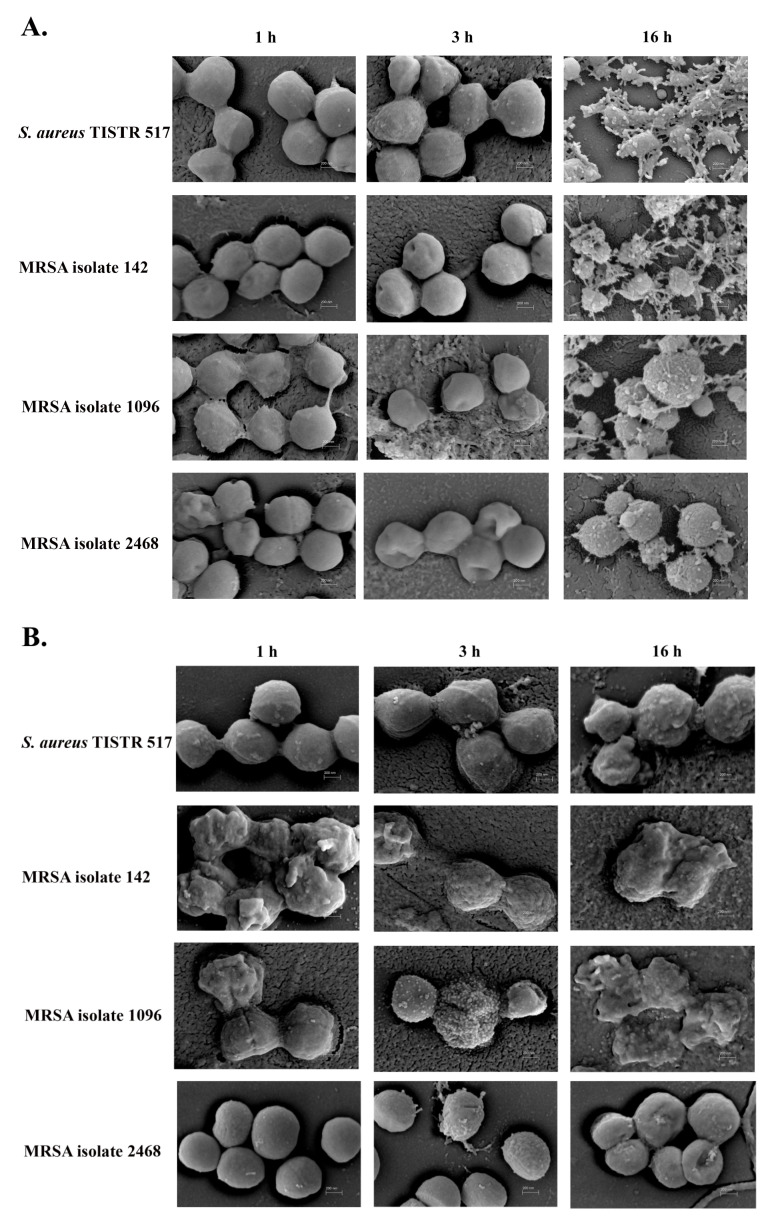

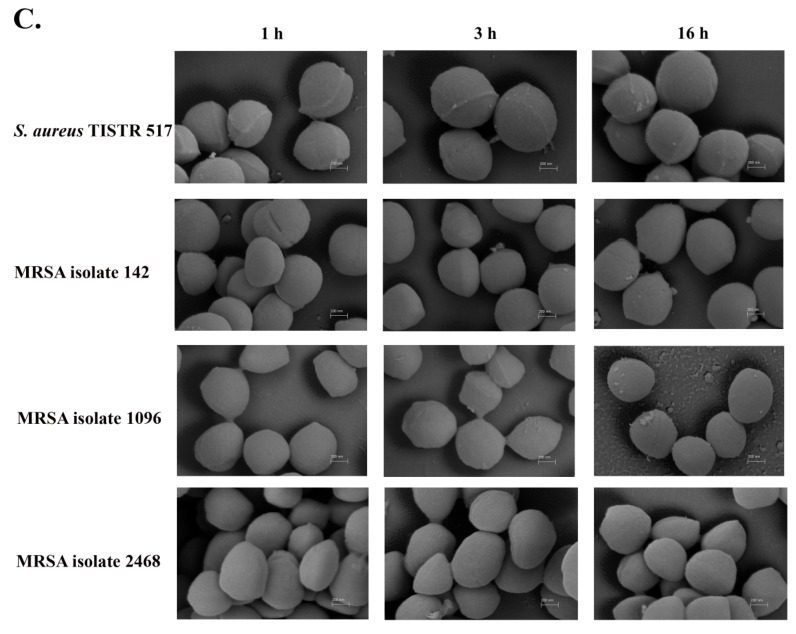
SEM micrograph of bacterial pathogens after the treatment of anti-MRSA peptide (P1). *S. aureus* TISTR 517 and MRSA isolates 142, 1096, and 2468 were treated with (**A**) P1 and (**B**) vancomycin at the 1× MIC level for 1, 3, and 16 h and compared to (**C**) the non-treatment condition.

The effect of P1 on bacterial pathogens was investigated by SEM. *S. aureus* TISTR 517 and all MRSA strains were damaged when treated with P1 and vancomycin. All bacterial membranes were sensitive to P1 at the 1× MIC level as judged by the rough and perforated surfaces that could not maintain their original shapes after the first hour of treatment. This circumstance was more apparent when the treatment duration was extended to 3 and 16 h (Figure 6A). Similarly, the bacterial membrane was impaired in the vancomycin treatment, resulting in cell lysis (Figure 6B). In addition, the cell membrane of the untreated bacteria had a round shape with a smooth surface throughout all incubations (Figure 6C). 

### 2.6. Stability Profiles of Anti-MRSA Peptide

The stability study showed that P1 was tolerant to heat exposure up to 100 °C and autoclave conditions. Its activity was insignificantly different from that of untreated samples, indicating that P1 was a thermostable peptide. The antimicrobial activity of vancomycin was significantly decreased when the temperature was raised above 80 °C (*p*-value < 0.05). Proteolytic enzymes are generally used to determine the degradation of AMPs. The result showed that all proteases caused a significant reduction in the antimicrobial activity of P1 (*p*-value < 0.05), confirming the presence of peptides in the structure. In addition, the activity of vancomycin was constant, reflecting its resistance to hydrolysis by those enzymes. Significantly increased P1 activity was observed when 1% SDS and Triton X-100 were included in the peptide solution (*p*-value < 0.05), indicating that P1 was unaffected by surfactants. Triton X-100 had no antimicrobial activity, whereas SDS showed an inhibitory effect. However, these surfactant conditions did not enhance the vancomycin activity. In addition, P1 resisted hydrolysis in a wide range of pH, and thus its remaining activity was not different from the untreated condition significantly. The activity of vancomycin considerably decreased when the pH of the sample was higher than 11. Collectively, the P1 activity remained over 90% under several stress conditions, indicating it was a highly stable peptide (Table 6).

### 2.7. Killing Kinetics of Anti-MRSA Peptide

**Figure 7:**
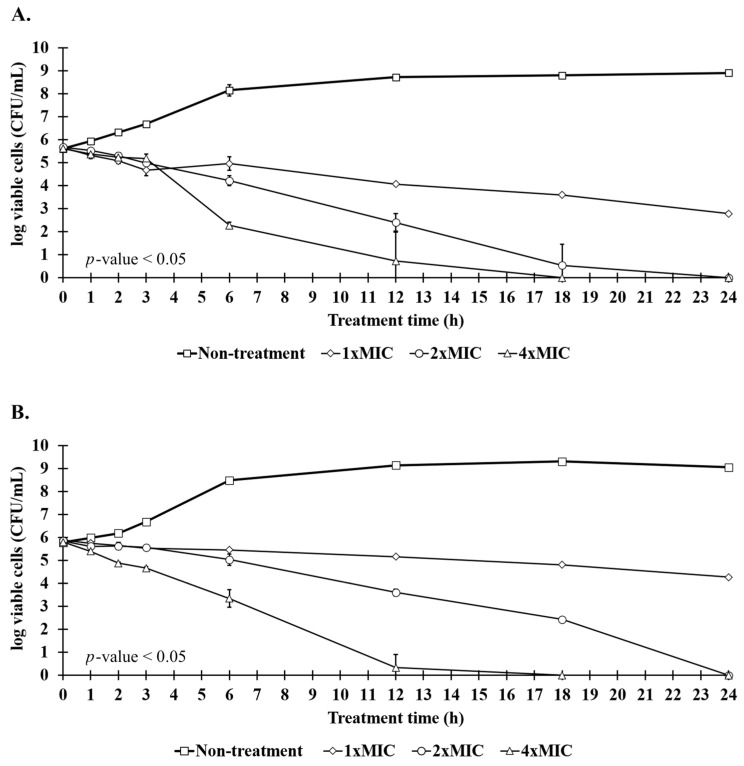
Killing kinetics of anti-MRSA peptide (P1). (**A**) *S. aureus* TISTR 517 and (**B**) MRSA isolate 2468 were incubated with 1× (◊), 2× (○), and 4× MIC (Δ) of P1 and compared to the non-treatment condition (□). The viable cells were counted by spreading an aliquot of samples on MH agar, and the result was expressed as a log of the viable cell count (CFU/mL). Significant differences (*p*-value <0.05) were analyzed by two-way ANOVA comparing treated and non-treated samples.

The reduction in the number of viable pathogens caused by P1 was determined at 1×, 2×, and 4× MIC. In the case of *S. aureus* TISTR 517, it was found that the reduction rates of all peptide concentrations were 0.14 h^−1^ in the first 3 h of treatment. The rates were different between 3 and 24 h of incubation, during which the reduction rates were 0.12, 0.23, and 0.31 h^−1^ for 1×, 2×, and 4× MIC, respectively (Figure 7A). Furthermore, a similar reduction rate of MRSA isolate 2468 in the first 3 h (0.10 h^−1^) was observed, and the inhibition rate was different after 6 h of treatment with 1× and 2× MIC of P1, being 0.06 and 0.24 h^−1^, respectively (Figure 7B). At the higher P1 concentration (4× MIC), the inhibition rate was increased with the initial rate of 0.38 h^−1^ after 3 h of treatment, and the rate was slightly decreased to 0.32 h^−1^ during 3–12 h. When compared to the cell numbers in the non-treatment condition, P1 at all concentrations significantly showed bacterial killing during 1–24 h (*p*-value < 0.05). P1 at 4× MIC demonstrated a significant killing effect after 6 h of incubation which was higher than that of 2× and 1× MIC (*p*-value < 0.05). Interestingly, P1 exhibited an eradication effect at 24 and 18 h for the peptide concentrations of 2× and 4× MIC, respectively, whereas the inhibition of bacterial growth was observed at 1× MIC. The rate constant of cell death within 24 h and the killing half-time were calculated based on linear regression. It was estimated that P1 had rate constants of −0.11 (95% CI −0.08 to −0.13), −0.26 (95% CI −0.23 to −0.29), and −0.27 h^−1^ (95% CI −0.16 to −0.38) for 1×, 2×, and 4× MIC, respectively, for *S. aureus* TISTR 517. The killing half-times were 25.36 (95% CI 19.77 to 34.10), 11.08 (95% CI 9.18 to 13.53), and 9.80 h (95% CI 5.35 to 20.12) for 1×, 2×, and 4× MIC, respectively, for *S. aureus* TISTR 517. Furthermore, the rate constants of P1 against MRSA isolate 2468 were −0.06 (95% CI −0.05 to −0.07), −0.23 (95% CI −0.19 to −0.27), and −0.27 h^−1^ (95% CI −0.17 to −0.36), whereas the killing half-times were 47.75 (95% CI 41.40 to 56.12), 13.21 (95% CI 10.42 to 17.15), and 9.78 h (95% CI 5.67 to 18.47) for 1×, 2×, and 4× MIC, respectively. These results demonstrated that P1 had bactericidal activity in both time- and concentration-dependent manners. 

### 2.8. Bacteriolysis and Leakage of Genetic Materials

**Figure 8:**
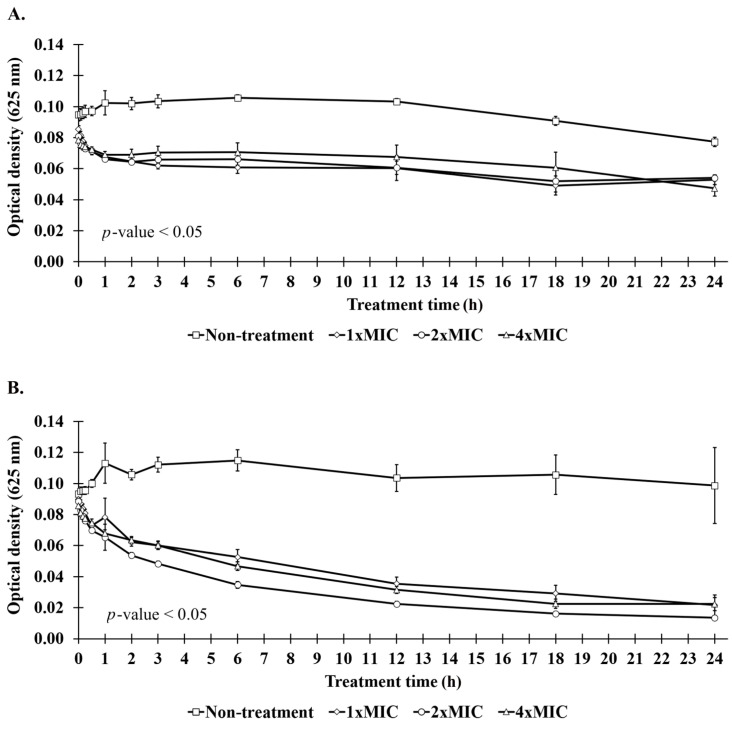

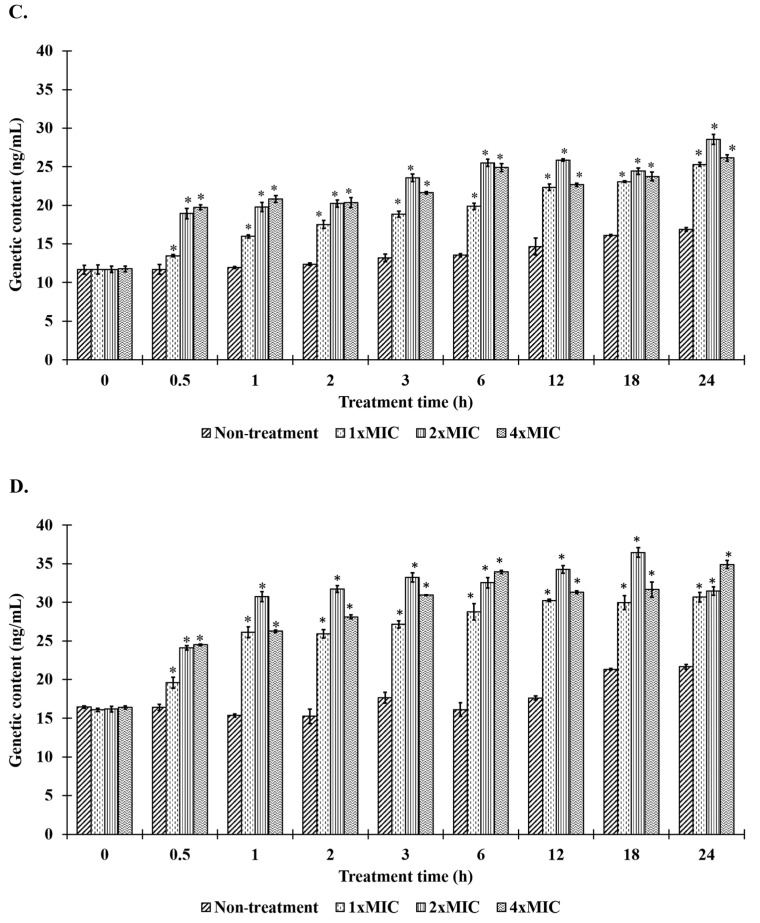
Effect of P1 on bacteriolysis. The bacterial cultures of (**A**) *S. aureus* TISTR 517 and (**B**) MRSA isolate 2468 in PBS supplemented with incomplete media (4% CAMHB) were incubated with the peptide samples (1×, 2×, and 4× MIC). The OD at 625 nm was monitored at different time intervals. The content of genetic leakage from (**C**) *S. aureus* TISTR 517 and (**D**) MRSA isolate 2468 was assessed by determining the absorbance at 260 nm. Significant differences (*) were analyzed by two-way ANOVA at *p*-value <0.05 compared to the non-treatment condition.

The induction of cell lysis by AMPs could be determined by measuring the optical density (OD) reduction at 625 nm because of cell breaks. The result showed that the OD of *S. aureus* TISTR 517, when incubated with P1, was rapidly decreased in the first hour of the treatment, in which the bacterial cells were destroyed at rates of 21.08 ± 1.11%, 18.24 ± 1.70%, and 12.00 ± 2.72% at 1×, 2×, and 4× MIC, respectively. The OD was constant between 0.047 and 0.054 at 24 h, indicating that the peptide caused the cell lysis of more than 39.67% (Figure 8A). A similar OD reduction within the 1 h of treatment was observed in MRSA isolate 2468 with the cell lysis of 12.68 ± 13.57%, 26.13 ± 9.48%, and 20.75 ± 2.75% when treated with P1 at 1×, 2×, and 4× MIC, respectively. The OD of the MRSA culture decreased to 0.014–0.022 at 24 h of incubation, indicating that at least 75.85% of cells were lysed (Figure 8B). These results revealed that P1 significantly caused bacterial lysis when compared to the non-treatment condition (*p*-value < 0.05). The leakage of genetic materials upon the membrane disruption was followed by measuring the increased absorbance at 260 nm of the cell supernatant incubated with AMPs. The result showed that the release of genetic materials was time-dependent; i.e., more genetic leakage was observed when the incubation time was longer. A significant increase in genetic leakage in *S. aureus* TISTR 517 and MRSA isolate 2468 was found at 30 min as the genetic content in the supernatant was increased by 1.09–1.60 and 1.15–1.47 times, respectively (*p*-value < 0.05) (Figure 8C,D). At the end of the treatment (24 h), the genetic release was dramatically increased by 2.05–2.29 and 1.81–2.07 times. Remarkably, these results did not allow discriminating whether the peptide (P1) effects of cell lysis and genetic leakage were dose-dependent. We subsequently counted the viable cells of the samples at 24 h and found that *S. aureus* TISTR 517 was inhibited by 8.60 ± 0.26%, 67.63 ± 1.96%, and 94.35 ± 9.78% when incubated with P1 at 1×, 2×, and 4× MIC, respectively. Meanwhile, the inhibition of MRSA isolate 2468 was 34.46 ± 0.64%, 64.47 ± 3.56%, and 96.13 ± 6.70% at 1×, 2×, and 4× MIC of P1, respectively. Although the result of bacteriolysis showed an incomplete cell break, it was confirmed that P1 could inhibit the bacterial pathogens in a concentration-dependent manner. This might implicate that other inhibitory mechanisms could contribute to P1 activity.

### 2.9. Effect of the Anti-MRSA Peptide on Cell Permeability

**Figure 9:**
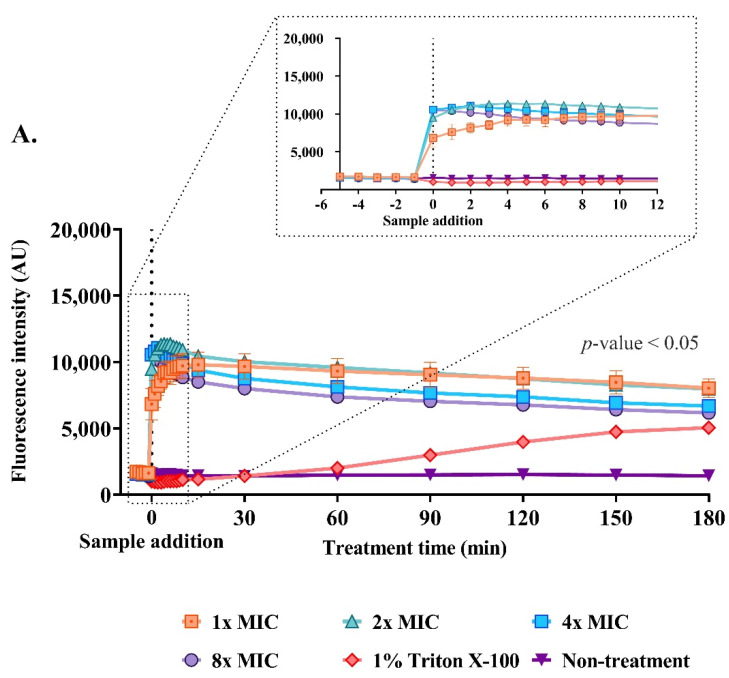

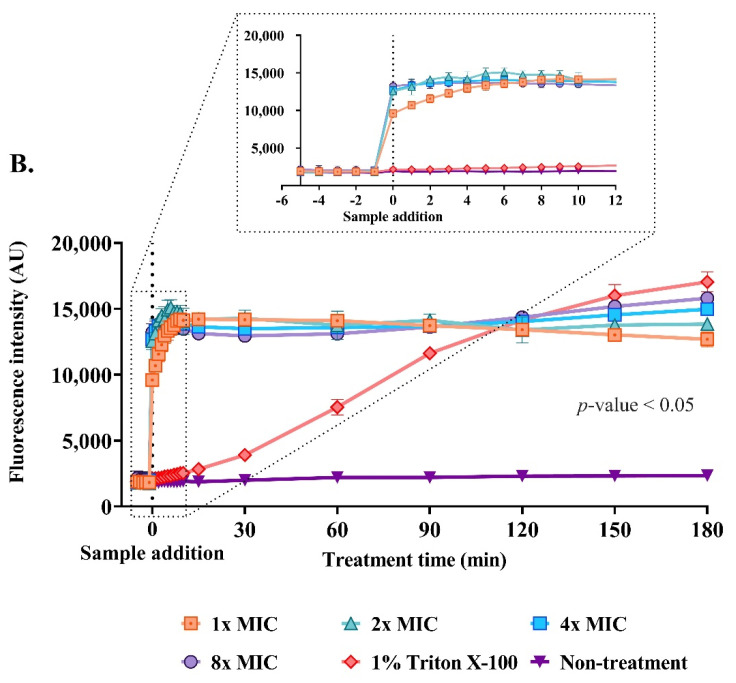
Effect of anti-MRSA peptide (P1) on membrane permeability. The cell cultures of (**A**) *S. aureus* TISTR 517 and (**B**) MRSA isolate 2468 were incubated with different concentrations of P1 (1×, 2×, 4×, and 8× MIC). Sytox green uptake was determined by measuring the fluorescence intensity at 532 nm in specific time intervals (0, 2.5, 5, 10, 15, 30, 60, 90, 120, 150, and 180 min). The inset graph shows the expanded view of the boxed region. The statistical significance of fluorescence intensity was determined between treated and non-treated samples (*p*-value < 0.05).

The penetration of an impermeable dye (sytox green) through the bacterial membrane is a characteristic of compromised cells. The interaction between the dye and nucleic acid results in an intense fluorescence, reflecting the occurrence of cellular permeability after the AMP function. Before the addition of P1 and Triton X-100, the fluorescence intensity in the bacterial cells was not significantly different from that of the non-treatment condition. An enormous increase in fluorescence was observed immediately after the P1 addition. The intensity after peptide addition and at the end of the study in *S. aureus* TISTR 517 was changed from 4.32 ± 0.85 to 6.95 ± 0.91, 5.62 ± 0.18 to 7.71 ± 0.11, 4.70 ± 0.16 to 7.38 ± 0.05, and 4.35 ± 0.16 to 7.06 ± 0.18 folds upon treatment with 1×, 2×, 4×, and 8× MIC of P1, respectively, when compared to the non-treatment condition (Figure 9A). Interestingly, higher fluorescence was observed in MRSA isolate 2468, indicating P1 caused more cell membrane permeability in MRSA isolate 2468 than in *S. aureus* TISTR 517. The intensity was elevated from 5.05 ± 0.46 to 7.58 ± 0.47, 5.92 ± 0.45 to 8.06 ± 0.71, 6.10 ± 0.25 to 7.54 ± 0.31, and 5.95 ± 0.30 to 7.46 ± 0.41 folds upon incubation with P1 at 1×, 2×, 4×, and 8× MIC, respectively (Figure 9B). The treatment of both bacterial pathogens showed a significant increase in fluorescence (*p*-value < 0.05) when compared to the non-treatment condition, indicating that the P1 mechanism involves cellular permeability. Triton X-100 was used as a positive control, and the treatment of tested bacteria with Triton X-100 induced the uptake of sytox green in a time-dependent manner. The increase in the fluorescence intensity produced by Triton X-100 compared to the non-treatment was from 0.61 ± 0.06 to 3.57 ± 0.40 and 1.12 ± 0.05 to 7.30 ± 0.71 folds for *S. aureus* TISTR 517 and MRSA isolate 2468, respectively. Furthermore, the treatment of MRSA isolate 2468 with Triton X-100 showed a higher rate of fluorescence in the first 90 min, and this signal was comparable to that of peptide treatment at 120 min. The level of fluorescence in the treatment of *S. aureus* TISTR 517 with Triton X-100 was lower than that of the peptide incubation at the end of the study.

### 2.10. Effect of the Anti-MRSA Peptide on Hemolysis

**Figure 10:**
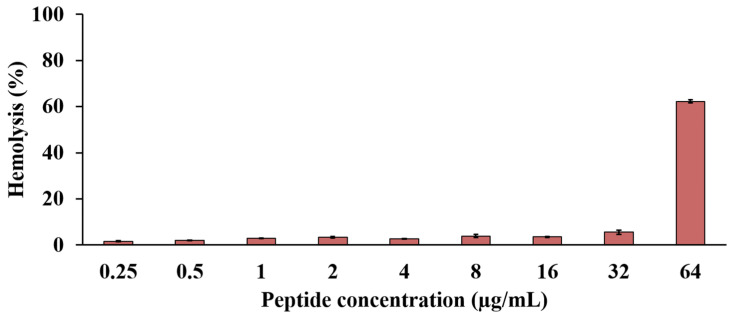
Lysis of human erythrocytes by the anti-MRSA peptide (P1). The peptide was prepared in concentrations between 0.25 and 64 µg/mL. The result is represented by % hemolysis, which was calculated by measuring the absorbance at 540 nm compared to 1% Triton X-100 as the positive control.

P1 exhibited hemolytic activity in a concentration-dependent manner. Degrees of hemolysis of less than 10% were observed in the peptide concentrations between 0.25 and 32 µg/mL and corresponded to hemolysis in the range of 1.64 ± 0.17% to 5.52 ± 0.92% (Figure 10). P1 at 1× MIC (2 µg/mL) showed hemolysis of 3.40 ± 0.29%. The highest hemolytic activity, 62.23 ± 0.69%, occurred upon incubation with 64 µg/mL of the peptide. The therapeutic index of hemolysis (TI_hemolysis_) was calculated as 16, reflecting a greater antimicrobial specificity of P1 in the in vitro study.

## 3. Discussion

The emergence of MRSA infection presently causes high mortality worldwide. It is one of the pathogens for which the search for novel antimicrobial agents is highly prioritized by the WHO. Soil bacteria are valuable resources for producing antibiotics to kill MRSA. Our study isolated SPR-20, which produced some antimicrobial substances that inhibited *S. aureus* TISTR 517 and MRSA strains. Bioassay-guided fractionation separated some anti-MRSA substances, and the final step of RPC could purify five active substances (P1–P5). These substances might have high hydrophobicity based on their elution by acetonitrile in the range of 26.36% to 38.25%. The de novo approach revealed the peptide sequences with a length of 14–15 residues. These AMPs were amphipathic molecules, containing the predicted isoelectric point between 8.62 and 9.93 with net positive charges (+1 to +3 at pH 7.4) and hydrophobicity (0.670 to 0.915). The presence of lysine (1–3 residues) in the peptides contributed to the positive charge of the molecules, whereas the high proportion of non-polar amino acids (10–13 residues), such as valine and leucine, provided lipophilicity that explained the higher concentration of acetonitrile in the mobile phase required for their elution in the RPC. 

Generally, the peptide elution from RPC depends on the sequence, ion-pairing agent, organic solvent in the mobile phase, and alkyl chain of the stationary phase [10]. Increasing the hydrophobic nature and concentration of the ion-pairing agents enhances the retention time and peak resolution of the peptides due to an overall increase in hydrophobicity of the peptide and ion-pairing agent complexes. This was demonstrated by the effect of various hydrophobic ion-pairing agents on the retention times of tested peptides (retention time of peptide with HFBA > PFPA > TFA > phosphate) [11]. In addition, the higher the positive charge of peptides, the longer the retention time (retention time of charge +5 > +3 > +1). This could be explained by an increase in the hydrophobicity of ion-pairing agents that were more masked by the greater positive charges on the peptides [11]. This is consistent with the results of our study, in which P1 (charge +1) was eluted faster than P2 (charge +2) and P3–P5 (charge +3), while P5 came later as its hydrophobicity was higher than that of P3 and P4, reflecting the influence of peptide charge and hydrophobicity on retention behavior. 

These peptides (P1–P5) were novel AMPs because they were not listed in the APD3 Antimicrobial Peptide Database (https://aps.unmc.edu/database; 3435 peptides; accessed on 25 August 2022) or the Database of Antimicrobial Activity and Structure of Peptides (DBAASP) (https://dbaasp.org/search; 19,316 peptides; accessed on 25 August 2022) [12,13]. In these two peptide datasets, there are 385 and 432 AMPs acquired from bacteria, respectively. Pairwise alignments using MatGAT (Matrix Global Alignment Tool) with BLOSUM62 scoring matrix showed that our anti-MRSA peptides (P1–P5) had the highest similarity (53–79%) to acidocin LCHV, bogorol, and brevilaterin analogs (Table 7) [14].

CD spectral analysis revealed that P1–P5 in water mostly had β-strand and disordered conformations. The β-branched amino acids (valine and isoleucine) are assigned as α-helix destabilizing residues, and they are favored to be in the β-strand [15]. The number of valine residues (4–8 residues) in our peptides promoted a considerable tendency to form β-strands. When these peptides were in the SDS solution, they (P1–P4) exhibited a structural change, resulting in the peptides had more folded α-helix and β-strand conformations that exerted antimicrobial activity. In contrast, the peptide P5 did not undergo structural alteration in the SDS solution, indicating that different modes of action contributed to its activity. Typically, AMPs contain secondary structures, such as α-helix (human cathelicidin LL-37), β-strand (human α-defensin 6), both α-helix and β-strand (human β-defensin 2), and disordered element (bovine indolicidin) structures [16]. When in close proximity to lipid membranes, they mainly cause conformational rearrangements that are associated with the change in hydrophobicity and cationic regions of the molecules and then exert antimicrobial activity through membrane disruption [7]. Several previous studies on CD spectra showed that AMPs, such as cecropin A and uperin 3, predominantly contained random coils in an aqueous solution, and the proportion of α-helical structure increased upon incubation with SDS (a membrane mimetic surfactant) [17,18]. The binding between the cationic residues of the peptides and the negatively charged sulfate on SDS facilitated the peptide interaction with the hydrophobic core of the SDS micelle. These circumstances increased the number of intramolecular hydrogen bonds, stabilizing the α-helical structure and reducing the intermolecular hydrogen bonds necessary for β-strands in peptides [18]. On the other hand, some antimicrobial peptides, such as indolicidin and OM19D analogs, had disordered structures in an aqueous solution, and their secondary structures did not change significantly in the SDS environment, indicating that other mechanisms of intracellular activity involved microbial inhibition rather than membrane disintegration [19,20].

The physicochemical properties, such as amphiphilicity, hydrophobicity, and secondary structure, affect the modes of action and potency of AMPs. One of the well-known mechanisms is the interaction between peptides and cell envelopes (cell wall and membrane) by which the positively charged peptides contact the negative charges of lipoteichoic acid or lipopolysaccharide of the bacterial membrane. The hydrophobicity of peptides supports the electrostatic interaction concomitant with the formation of α-helixes and the binding of the peptides to the membrane by hydrophobic interactions. Subsequently, the peptides destabilize the bacterial membrane by the barrel stave, toroidal pore, or carpet model [21]. Alternatively, some antimicrobial peptides can cross through the cellular membrane and function in intracellular activities, such as alterations in the synthesis of the cell wall, nucleic acids, and proteins or autolysis activation. The abovementioned mechanisms lead to bacteriolysis and cell death [22,23]. 

Our results showed the highest potency of P1 against *S. aureus* TISTR 517 and MRSA strains (MIC and MBC of 2 and 4 μg/mL, respectively). Furthermore, P1 (molecular mass 1570.02 g/mol) had greater inhibitory activity than vancomycin (molecular mass 1449.20 g/mol) as their MIC values were 1.27 and 1.38 μM, respectively. Other peptides (P2–P4) also exhibited activity against the tested bacteria with MIC and MBC values of 8–32 μg/mL and 4–32 μg/mL, respectively. MRSA strains were mostly killed at 2× MIC. The study of potency comparison showed that P1 was more potent than vancomycin based on the agar well diffusion method (5.44 and 6.45 times for *S. aureus* TISTR 517 and MRSA isolate 2468, respectively). The highest activity of P1 might be explained by its physicochemical property as a membrane-active peptide. Although P1 had the lowest positive charge, it had the highest hydrophobicity and contained three prolines in the sequence. The superior activity might result from the enhancement of cellular penetration of polyproline and the strong interaction of the peptide hydrophobic patch with the bacterial membrane [24]. Another characteristic of the polyproline-containing peptide was the formation of α-helix structures upon combination with SDS, which enhanced antimicrobial activity [25]. This phenomenon was consistent with our CD study, in which P1 exhibited an increased proportion of α-helical conformation when dissolved in the SDS solution. P3 and P4 (charge +3) had similar hydrophobicity, and they exhibited more killing activity than P2 (charge +2). The additional positively charged lysine at the carboxyl termini of P3 and P4 was postulated to directly bind to the cell membrane and then display more activity. This was also observed in other investigations in which the modification of peptide with lysine at both termini (3K-F17-4L-3K) resulted in greater antimicrobial activity than the original peptide that contained lysine at the amino terminus (6K-F17-4L) while both peptides had the same hydrophobicity [26]. Although P5 had the lowest activity, it still inhibited the tested bacteria, so its unchanged structure in both water and SDS might cause different potency. Taken together, these results indicate that effective antimicrobial activity is promoted by balancing the distribution of hydrophobicity and charge along with the structural adaptability of the peptides [21].

With the highest activity, P1 was selected for further study on its stability and mechanism of action. P1 was thermostable as it maintained nearly all activity after exposure to 100 °C or autoclave conditions. The short-chain peptide with substantial β-strand and disordered structures was an important characteristic of P1. This peptide might be refolded to active conformation after heat treatment that conferred good thermal stability. Similarly, acidocin LCHV (11 residues) produced by *Lactobacillus acidophilus* n.v. Er 317/402 Narine was stable at 130 °C for 90 min [27]. Brevilaterin V (11 residues) from *Brevibacillus laterosporus* S62-9 retained 99% of its initial activity after incubation at 100 °C for 2 h [28]. 

The acid–base environment can catalyze the hydrolysis and involves the different ionization states of AMPs that affect their stability and efficacy. However, the peptide solutions were neutralized before activity was determined in this study. Acidocin LCHV was stable at pH values from 3.0 to 6.5, but its activity was decreased at higher pH. The activity of Brevilaterins remained at 97% after incubation at pH 2.0–12.0. Similarly, P1 was stable over a broad pH range; its activity was unaffected when incubated at pH 2–14, showing its versatility for dosage formulations. 

Proteolytic enzymes are key factors in degrading peptides and diminishing peptide activity. P1 had an activity of more than 90% when incubated with these enzymes. The enzymatic stability of P1 was caused by the absence of aromatic amino acids, which are the specific cleavage sites of proteinase K and α-chymotrypsin. The presence of one lysine at the core sequence of P1 might lessen the sensitivity of trypsin [29]. According to a previous study, trypsin, pepsin, and proteinase K decreased the activity of acidocin LCHV. Brevilaterin V showed promising stability in response to pepsin, trypsin, and proteinase K with a remaining activity of more than 97% [28]. Interestingly, P1 might degrade more when exposed to proteolytic enzymes for a longer duration, which would reduce its residual impact on antimicrobial resistance in the microbial environment [30].

Surfactants are common chemicals used in drug preparation. Therefore, peptide stability in such excipients is helpful for formulation development. SDS is an anionic surfactant that can bind to a cationic peptide. The increase in SDS concentration was reported to attenuate the antimicrobial activity of AMPs [G-(IIKK)3I-NH_2_] with strong electrostatic interaction [31]. Triton X-100 is a nonionic surfactant that has been shown to increase the cellular permeability of AMPs. The susceptibility of melittin-resistant MRSA to melittin was found to be restored upon co-incubation with Triton X-100 [32]. Our study revealed that P1 activity significantly increased when SDS and Triton X-100 were included. The enhanced activity might result from membrane disruption by surfactants and structural change of P1 in the more α-helical conformation. Triton X-100 did not affect the P1 activity, suggesting that the nonionic nature was independent of peptide function.

P1 could induce perforation of the bacterial membrane in the first hour of treatment as judged by the blisters on the cell surface analyzed by SEM. Burst cells were found after prolonged incubation, indicating that the P1 mechanism of action involves membrane damage and disintegration. Vancomycin similarly triggered membrane poration and led to cell lysis. The drug binds D-ala-D-ala, which is a precursor of cell wall synthesis and then kills bacteria by altering cell permeability [33].

The decrease in OD at 625 nm can be used to measure bacterial lysis. Our study found a rapid OD reduction in the tested bacteria during the first hour of P1 treatment, suggesting that a significant amount of lysed cells was present because they produced less light scattering than intact cells. A gradual decrease in OD was observed after 2 h, but it never had a value of zero. This may be a drawback of the method by which the light is scattered by the living and dead cells. It is proposed that the peptide was thoroughly incorporated in most bacterial populations with incomplete lysis, and the cell debris still affected the OD value. We confirmed the killing effect of P1 by counting the viable cells after 24 h of investigation. It was found that the bacterial cells were destroyed by P1 in a time- and concentration-dependent manner.

The membrane disruption can be monitored by studying the leakage of genetic materials and the penetration of the impermeable fluorescent dye. After the addition of P1, the release of genetic materials was gradually enhanced based on absorbance at 260 nm, whereas the permeation of sytox green was rapidly allowed to intercalate the bacterial DNA, increasing the fluorescence intensity at 523 nm. These phenomena could be explained by the pore formation on the cellular membrane during peptide treatment [34]. In the initial period of P1 treatment, the membrane pores were so small that they could only allow the sytox green molecule to pass through and bind with bacterial chromosomes, which immediately increased the fluorescence signal. The signal was constant after 10 min of incubation, indicating the equilibrium of the dye–bacterial DNA complexes. The gradual leakage of genetic materials within 30 min was observed because bacterial chromosomes needed a larger pore to pass through the cell membrane and enter the extracellular solution [35]. Collectively, these results indicated that P1 targeted the bacterial membrane by altering cellular permeability and rupturing the membrane. 

A significant difference in the killing kinetics of P1 was found after incubation for 6 h. The reduction in viable cells greater than 3 logs exhibited its bactericidal effect. The killing effect was strongly associated with peptide concentration and incubation period, and P1 at 4× MIC completely eradicated the tested bacteria at 18 h. Several studies demonstrated that the activity of AMPs was dependent on concentration. Similar to P1, penisin from *Paenibacillus* sp. A3 displayed an incomplete killing at 1× MIC but enabled eradication above 2× MIC [36]. Acidocin LCHV was found to completely kill *S. aureus* in 10 min without regrowth of the bacterial cells.

Hemolysis is a primary standard for evaluating the safety of AMPs on eukaryotic cells. The peptide hydrophobicity is the primary factor that induces the lysis of erythrocytes. Higher hydrophobicity is associated with stronger hemolysis because an increased hydrophobicity facilitates the partition of a large proportion of peptides into the plasma membrane, causing membrane damage [37]. For example, Brevilaterin V2 had lower hydrophobicity than Brevilaterin A, and it had less hemolytic activity than Brevilaterin A (4.06 ± 0.84% vs. 13.41 ± 3.26% at 500 μg/mL) [28]. Our investigation revealed that the hemolysis of P1 was dependent on its concentration. The high hydrophobicity of P1 contributed to this effect. Its estimated TI_hemolysis_ was 16, indicating the antimicrobial specificity of P1 that supports its potential for clinical use [38]. Interestingly, some studies showed that the peptide hydrophobicity beyond an optimum value reduced the antimicrobial activity as the peptide self-association occurred in the aqueous solution and prevented its penetration into bacterial cell walls [39]. This indicates that the balance of peptide hydrophobicity and hydrophilicity can maximize antimicrobial activity and reduce hemolysis.

In summary, P1 exhibited the highest anti-MRSA activity in this study. Its activity was concentration- and time-dependent, and the mechanism of action involved membrane disruption and eventual cell lysis. This peptide demonstrated great stability in response to temperature, surfactants, pH, and enzymes. P1 was not harmful to human erythrocytes. It is beneficial for peptide formulation in the pharmaceutical field. However, it requires further investigations, elucidating its exact molecular targets and cytotoxicity against mammalian cells. These novel peptides provide an opportunity to treat MRSA infection.

## 4. Materials and Methods

### 4.1. Bacterial Strains and Culture Condition

*Brevibacillus* sp. SPR-20 (GenBank accession number MN533919) was cultured on MH agar (Titan Biotech Ltd., Rajasthan, India) at 30 °C for 24 h. A single colony was resuspended in 0.85% NaCl, and the optical density (OD) at 625 nm was adjusted to 0.1 for preparation of the initial cell suspension. A standard strain of *S. aureus* TISTR 517 was purchased from the Thailand Institute of Scientific and Technological Research (TISTR), Thailand. MRSA isolates 142, 1096, and 2468 were from clinical specimens from Maharaj Nakhon Si Thammarat Hospital, Nakhon Si Thammarat, Thailand.

### 4.2. Purification of Anti-MRSA Substances

Eight milliliters of the SPR-20 starter culture was added to 392 mL of Luria–Bertani (LB) broth (Titan Biotech Ltd., Rajasthan, India) in a 2 L flask. The culture was incubated at 30 °C and 150 rpm for 24 h before the crude supernatant was collected by centrifugation at 10,000× *g* and 4 °C for 15 min. Ammonium sulfate at 75% of saturation was added to the supernatant, and the precipitate was desalted by dialysis using a 3.5 kDa molecular weight cut-off (MWCO) SnakeSkin membrane (Pierce, Rockford, IL, USA) in the dialysis buffer (50 mM ammonium acetate pH 5.0 and 50 mM NaCl) at 4 °C for 16 h. The sample solution was purified by a HiTrap SP column under the elution gradient of buffer A (50 mM ammonium acetate pH 5.0 and 50 mM NaCl) and buffer B (50 mM ammonium acetate pH 5.0 and 1 M NaCl). The flow rate was set at 3 mL/min, and the wavelength was monitored at 214 nm. The active fractions were subjected to reversed-phase chromatography (RPC) by using an Inertsil ODS-3 C18 column (4.6 × 250 mm; GL Sciences, Tokyo, Japan). A gradient of buffer A (0.1% trifluoroacetic acid; TFA in water) and buffer B (0.1% TFA in 90% acetonitrile; ACN) at a flow rate of 1 mL/min was used for elution with the following conditions: 0.0% buffer B for 20 min, 0.0% to 29.3% buffer B for 10 min, 29.3% to 42.5% buffer B for 90 min, 42.5% to 66.7% buffer B for 10 min, and 66.7% to 100% buffer B for 10 min. The peak was detected at 214 nm. Each fraction was subsequently evaporated in a speed vacuum concentrator, and the dried substance was dissolved in 0.85% NaCl before an agar well diffusion assay was performed. The active fractions obtained from each purification step were prepared at different concentrations using 2-fold dilution, and the antimicrobial activity against MRSA isolate 2468 was determined by agar well diffusion assay. The arbitrary activity was calculated by the following equation [40,41]:

(1) $$\mathrm{Activity}\;(\mathrm{AU}/\mathrm{mL})=\frac{2^{n}\times \;1000}{V}$$

where n is the number of the highest dilution that showed the inhibitory zone and v is the volume of sample in microliters used in each well.

### 4.3. Microdilution Antimicrobial Assay

The MIC and MBC of the purified peptides from SPR-20 were determined by broth microdilution assay following a standard guideline [42]. *S. aureus* TISTR 517 and MRSA isolate 142, 1096, and 2468 as bacterial strains were cultured in MH agar at 37 °C for 18 h. A single colony of tested bacteria was suspended in 0.85% NaCl until its turbidity had an OD of 0.1 at 625 nm. The cells were then diluted to 5 × 10^6^ CFU/mL using cation-adjusted Mueller Hinton broth (CAMHB). The diluted cell suspension (10 μL) was transferred to each well of a 96-well plate. The purified peptide in the total volume of 100 µL was added to each well to obtain the final concentrations between 0.5 and 64 µg/mL. The standard antibiotics (vancomycin and cefoxitin) (Sigma-Aldrich Co., St. Louis, MO, USA) and antibiotic-free samples were the positive and negative controls, respectively. CAMHB without tested bacteria was used as a blank. The 96-well plates were incubated at 37 °C for 24 h, and the experiment was performed in triplicate for each strain. The MIC was evaluated using the lowest concentration of the substances that showed no observable growth of bacteria. Subsequently, 100 µL of each dilution was spread on MH agar and then incubated at 37 °C for 24 h. The lowest concentration of the substances that showed no colony was defined as the MBC [43].

### 4.4. Agar Well Diffusion Assay

The antimicrobial activity was measured by an agar well diffusion test with some modified methods [44]. In brief, single colonies of the tested strains were suspended in 0.85% NaCl, and then the OD at 625 nm was adjusted to 0.1. The cell suspensions were spread on MH agar, and the tested samples (100 μL) were introduced into each well before incubation at 37 °C for 24 h. Each experiment was performed in three independent replicates, and the inhibition zone was measured and expressed as mean ± SD. 

### 4.5. Peptide Sequencing

An UltiMate 3000 liquid chromatography (LC) system combined with a high-resolution mass spectrometer was used for peptide sequencing (Thermo Fisher Scientific Inc., Waltham, MA, USA). The peptide solution in 0.1% formic acid and 1% ACN was loaded on an analytical C18 column, and the peptide was separated by using a 40 min gradient of buffer A (0.1% formic acid) and buffer B (0.1% formic acid in ACN) at the flow rate of 300 μL/min. For this LC setup, the sample (3 μL) was directly injected into the LC system, and a blank (0.1% formic acid) was run between samples to prevent sample carry-over. The protonated peptides were transferred to an ESI source of LC-MS/MS by using the capillary voltage (3.2 kV) at 300 °C. To obtain a full-length peptide sequence by de novo sequencing, the combination of LC-MS data with full MS scanning and target list scanning was performed at the same LC conditions. Briefly, the LC-MS/MS was operated in full MS scanning mode. The parameters were as follows: MS resolution, 120,000; automatic gain control (AGC) target, 1 × 10^6^; maximum injection time (IT), 100 ms; scan range, *m*/*z* 400–2200. Full MS scan LC-MS files were processed using Freestyle software (version 1.4) (Thermo Fisher Scientific Inc., Waltham, MA, USA). Automatically detected chromatographic peaks were applied using the PPD algorithm. The specific absolute signal-to-noise threshold was set at 5. The identified peaks were used in the mass inclusion list in the parallel reaction monitoring (PRM) mode. To obtain the full ion spectrum of each identified peak in the inclusion list, the samples were subjected to LC-MS/MS again in PRM mode. The parameters were as follows: MS2 resolution, 30,000; AGC target, 5 × 10^5^; maximum IT 150 ms; isolation windows, *m*/*z* 1.4. The PRM LC-MS files were processed using Peak Studio X (Bioinformatics Solutions Inc., Waterloo, CA, USA) for peptide sequencing [45,46]. The possible peptide sequence with a score of >70 and a level of confidence of >70 was acceptable. The physicochemical properties (peptide mass, hydrophobicity, isoelectric point (pI), and net charge at pH 7.4) of the identified peptides were predicted by HeliQuest [47].

### 4.6. Determination of Peptide Secondary Structure 

The purified peptides (0.6 mg/mL) were dissolved in deionized water or 50 mM SDS and introduced into the circular dichroism (CD) spectrophotometer (JASCO Corporation, Tokyo, Japan) [48]. A 1 mm quartz cell was used for the measurement. The CD spectra were collected at 25 °C in the wavelength between 190 and 260 nm with a scanning speed of 100 nm/min, a bandwidth of 1.0 nm, and a data resolution of 0.1 nm. Each experiment was performed in three replications, and the background spectrum of the corresponding solvent was subtracted from the peptide spectrum. The content of the secondary structure of the peptides was calculated using the CONTINLL program [49].

### 4.7. Sodium Dodecyl Sulfate–Polyacrylamide Gel Electrophoresis (SDS-PAGE) and Gel Overlay Assay

The active fractions (5 μg) were loaded in duplicate lanes in a 12% SDS-PAGE gel. A protein marker (Vivantis, Shah Alam, Selangor Darul Ehsan, Malaysia) was also used in this gel for size comparison. The gel was immersed in Tris-glycine buffer pH 8.3 and electrophoresed at 100 V. A half gel was excised and visualized by silver staining to detect the protein band. Another half gel was fixed with a solution of 25% methanol and 5% glacial acetic acid for 30 min and then washed in purified water for 3 h. The gel was overlaid by soft MH agar that contained MRSA isolate 2468 (10^6^ CFU/mL). The overlaid gel was then incubated at 37 °C for 24 h to monitor the position of the inhibition zone [50].

### 4.8. Potency Comparison between Anti-MRSA Peptide and Antibiotic

The potency of the purified anti-MRSA peptide was compared with that of vancomycin [51,52]. Vancomycin standard was prepared in 5 concentrations (50–150 μg/mL), and the anti-MRSA peptide (15 μg/mL) was dissolved in purified water. The agar well diffusion test was performed in triplicate for potency determination, using *S. aureus* TISTR 517 and MRSA isolate 2468 as the tested bacteria. The standard and sample solutions (100 μL) were added to each well, and the standard solution of the median concentration (100 μg/mL) was loaded into 3 wells in every plate for potency comparison. The remaining standard and sample solutions were transferred to the other 3 wells. The plates were incubated at 37 °C for 18 h. The zone of inhibition was measured, and its value was corrected for the variation among plates by normalizing the diameter of the inhibition zone of the median vancomycin concentration within and across all plates. The corrected zone of inhibition was plotted against the vancomycin concentrations, and the equivalence potency of the anti-MRSA peptide was determined from this calibration curve. The comparison of the inhibition zone between P1 and vancomycin was analyzed by Student’s *t*-test at *p*-value < 0.05.

### 4.9. Scanning Electron Microscopy (SEM) of Antimicrobial Peptide-Treated Cells

The SEM micrograph method was that of a previous study with some modifications [53]. The bacterial pathogens were incubated with anti-MRSA peptide from SPR-20 or vancomycin at the 1× MIC level for 1, 3, and 16 h before being fixed on a glass slide with 2.5% glutaraldehyde in 0.1 M phosphate buffer pH 7.2 for 24 h. The treated pathogens were dehydrated using a step gradient of ethanol from 20% to 100%, and then ethanol was evaporated by critical point drying (Quorum Technologies Ltd., Lewes, East Sussex, UK). The dried samples were coated with a gold sputter and visualized by SEM (Carl Zeiss, Oberkochen, Germany) at 50,000× magnification.

### 4.10. Stability Study of Anti-MRSA Peptide

The stability of the anti-MRSA peptide against several conditions was studied [54]. The sample solution (15 μg/mL) was subjected to 60, 80, and 100 °C for 1 h and 121 °C for 15 min (autoclave condition). The sensitivity of the peptide sample to proteolytic enzymes was investigated by incubating the sample with proteinase K, trypsin, and α-chymotrypsin (1 mg/mL) for 1 h. Surfactant compatibility of the substance was examined by incubating the sample with 1% SDS and 1% Triton X-100 for 1 h (AppliChem GmbH, Darmstadt, Germany). The acid and base hydrolysis property of the substance was assessed by pH adjustment to 2–14 for 1 h before the samples were neutralized to pH 7. After all treatments, the antimicrobial assay by agar well diffusion against *S. aureus* TISTR 517 and MRSA isolate 2468 was performed in three independent experiments. The stability profiles were presented as the percentage of activity relative to the untreated condition (mean ± SD), and significant difference was determined by the Student’s *t*-test at a *p*-value < 0.05. Vancomycin (300 μg/mL) was used as the standard for stability comparison.

### 4.11. Killing Kinetics of Anti-MRSA Peptide

*S. aureus* TISTR 517 and MRSA isolate 2468 were cultured in CAMHB and standardized to obtain the cell number of 5 × 10^5^ CFU/mL for initial treatment. The concentrations of peptides were prepared by diluting their concentrations to 1×, 2×, and 4× MIC by CAMHB, whereas the peptide-free CAMHB was used for non-treatment conditions. The reactions were incubated at 37 °C, and then the total volume was spread on MH agar at specific time intervals (0–24 h). The plates were incubated at 37 °C for 24 h before colony count. The trends in bacterial reduction were displayed by a log10 scale of cell number against the incubation time. The rate constant of cell death within 24 h and the killing half-time were calculated based on slope and linear regression relationship, respectively. Each experiment was performed in triplicate in 96-well plates [35]. Significant differences (*p*-value < 0.05) were analyzed by two-way ANOVA and post hoc Tukey’s test for multiple comparisons between treated and non-treated samples. 

### 4.12. Cell Lysis and Release of the Genetic Materials

The lysis of bacterial cells was monitored by measuring the OD reduction at 625 nm, whereas the leakage of genetic material was evaluated by measuring the absorbance at 260 nm [35]. Briefly, the bacterial colonies were collected from MH agar and then washed with PBS pH 7.4 three times. The OD at 625 nm of the washed cells was adjusted to 0.35 with PBS pH 7.4 supplemented with an incomplete medium (4% CAMHB) before the cells were transferred to a 96-well plate (100 μL/well). The stock solution of the anti-MRSA peptide was diluted with PBS pH 7.4, and 100 μL of the diluted samples was added to each well to obtain the final peptide concentrations of 1×, 2×, and 4× MIC. For each treatment condition at different time intervals, the reduction in OD at 625 nm was determined, and the supernatant of the treated samples was collected to measure the absorbance at 260 nm. The experiment was performed in triplicate, and the results were expressed as mean ± SD. Two-way ANOVA and post hoc Tukey’s test (*p*-value < 0.05) were used to determine the significance of differences between treated and non-treated samples. 

### 4.13. Effect of the Anti-MRSA Peptide on Membrane Permeability

The overnight cultures (16 h) of *S. aureus* TISTR 517 and MRSA isolate 2468 were collected and washed with sterile PBS pH 7.4 supplemented with 0.2% CAMHB as a diluent three times. The cell suspension was diluted by the diluent until the OD at 625 nm was equal to 0.1 (1 × 10^8^ CFU/mL). An aliquot of the diluted cells (100 μL) was introduced into each well and incubated with 10 μM sytox green (Thermo Fisher Scientific Inc., Waltham, MA, USA) in a dark place for 15 min. The anti-MRSA peptide was diluted with a diluent before 100 μL was placed in the wells to acquire the final concentrations of 1×, 2×, 4×, and 8× MIC. After the introduction of the anti-MRSA peptide (0–180 min), the degree of membrane permeability was determined by measuring the fluorescence intensity using a microplate reader (Thermo Fisher Scientific Inc., Waltham, MA, USA) with the excitation and emission wavelength at 504 and 523 nm, respectively. Significant differences in fluorescence intensity (*p*-value < 0.05) were analyzed by two-way ANOVA and post hoc Tukey’s test for multiple comparisons between treated and non-treated conditions. 

### 4.14. Effect of the Anti-MRSA Peptide on Hemolysis

Human blood (5 mL) from a healthy volunteer was collected in an EDTA tube. The erythrocytes were centrifuged at 700× *g* for 8 min and washed with PBS pH 7.4 three times. The erythrocytes were diluted with PBS pH 7.4 to reach a 0.5% *v*/*v* concentration. The suspension of erythrocytes (50 μL) was transferred to each well in a 96-well plate. The anti-MRSA peptide was dissolved and prepared by performing a 2-fold serial dilution with PBS pH 7.4 before the samples (50 μL) were added to the wells to achieve the final concentrations of 0.25–64 μg/mL. The reactions were incubated at 37 °C for 1 h, and the supernatant was collected by centrifugation at 1000× *g* for 10 min. The release of hemoglobin was monitored by measuring the absorbance at 540 nm. PBS pH 7.4 and 1% Triton X-100 were used as the negative and positive controls, respectively. The relative hemolysis was determined by normalizing the absorbance of the sample by that of the positive control. Each experiment was performed in three replications. The TI_hemolysis_ of the anti-MRSA peptide was calculated by dividing the highest concentration of the active peptide that produced hemolysis of less than 10% by its MIC value [55,56].

## 5. Conclusions

Five novel anti-MRSA peptides were isolated from *Brevibacillus* sp. SPR-20. These peptides showed inhibition against *S. aureus* and MRSA strains. P1 (NH_2_-VVVNVLVKVLPPPVV-COOH) exhibited the highest activity among the purified peptides and had higher potency than vancomycin. Structural analysis revealed that this peptide contained β-strand and disordered conformations, and the proportion of α-helical structure was increased after incubation with SDS. The mode of P1 action involved membrane leakage and cell rupture that killed MRSA in a peptide concentration- and treatment time-dependent manner. P1 was compatible with erythrocytes with a TI_hemolysis_ of 16. These findings might lead to the discovery of potential anti-MRSA peptides, and this could be an essential piece of antibiotic discovery for treating drug-resistant bacteria.

## Figures and Tables

**Table 1:** The purification balance sheet of anti-MRSA substance (P1) from SPR-20. The antimicrobial activity was determined by an agar well diffusion test against MRSA isolate 2468.

Purification Step	Volume (mL)	Total Protein (mg)	Total Activity (AU)	Specific Activity (AU/mg)	Purification Factor	%Yield
Cell-free supernatant	1740	446	69,600	156	1	100
Ammonium sulfate precipitation	205	231	65,600	284	2	94
CIEX	277	49	22,160	452	3	32
RPC (P1)	23	9	14,720	1694	11	21

**Table 2:** The MIC and MBC values of the purified substances from SPR-20. The active substances from RPC were used to determine the antimicrobial activity. The standard antibiotics (vancomycin and cefoxitin) were used as the positive controls.

Active Substance	Tested Strains	MIC (µg/mL)	MBC (µg/mL)
P1	*S. aureus* TISTR 517	2	4
	MRSA isolate 142	2	4
	MRSA isolate 1096	2	4
	MRSA isolate 2468	2	4
P2	*S. aureus* TISTR 517	8	16
	MRSA isolate 142	8	64
	MRSA isolate 1096	8	64
	MRSA isolate 2468	8	64
P3	*S. aureus* TISTR 517	4	4
	MRSA isolate 142	4	8
	MRSA isolate 1096	4	8
	MRSA isolate 2468	4	8
P4	*S. aureus* TISTR 517	8	8
	MRSA isolate 142	8	16
	MRSA isolate 1096	8	16
	MRSA isolate 2468	8	16
P5	*S. aureus* TISTR 517	32	32
	MRSA isolate 142	32	32
	MRSA isolate 1096	32	32
	MRSA isolate 2468	32	32
Vancomycin	*S. aureus* TISTR 517	2	2
	MRSA isolate 142	2	2
	MRSA isolate 1096	2	2
	MRSA isolate 2468	2	2
Cefoxitin	*S. aureus* TISTR 517	2	2
	MRSA isolate 142	>64	ND
	MRSA isolate 1096	>64	ND
	MRSA isolate 2468	>64	ND

**Table 3:** Physicochemical properties of anti-MRSA peptides from SPR-20. The peptide sequences were obtained from de novo sequencing by LC-MS/MS. The predicted mass, pI, net charge, and hydrophobicity were calculated.

Sample	Sequence (N→C)	Length (Residues)	Mass (Da)	pI	Net Charge at pH 7.4	Hydrophobicity (H)
P1	VVVNVLVKVLPPPVV	15	1570.02	8.62	+1	0.915
P2	VVMNLLVKVLKYVV	14	1616.01	9.51	+2	0.859
P3	VVLNLLVKVLKYGK	14	1585.03	9.93	+3	0.648
P4	VVVNLLVKVLKYAK	14	1585.03	9.93	+3	0.636
P5	VVLNLVVKLLKYAK	14	1599.05	9.93	+3	0.670

**Table 4:** Estimation of secondary structure content of anti-MRSA peptides. The peptides were dissolved in the different solvents (deionized water and 50 mM SDS), and the predicted secondary structures were analyzed by the CONTINLL program.

Peptide	Solvents	α-Helix (%)	β-Strand (%)	Turn (%)	Random (%)	Total (%)
P1	H_2_O	4.1	44.0	19.5	32.4	100.0
	50 mM SDS	25.5	29.3	10.6	34.6	100.0
P2	H_2_O	3.4	39.8	20.5	36.3	100.0
	50 mM SDS	3.7	48.8	20.8	26.7	100.0
P3	H_2_O	3.9	40.3	22.6	33.2	100.0
	50 mM SDS	12.4	29.0	10.6	48.0	100.0
P4	H_2_O	3.5	42.0	20.8	33.7	100.0
	50 mM SDS	4.9	38.7	22.7	33.7	100.0
P5	H_2_O	3.1	43.1	21.2	32.6	100.0
	50 mM SDS	2.6	40.7	19.3	37.4	100.0

**Table 5:** Potency comparison between anti-MRSA peptide (P1) and vancomycin. The inhibition zones of P1 and vancomycin in the agar well diffusion test were measured, and the results are presented as mean ± SD.

Concentration	Corrected Zone of Inhibition (Mean ± SD; mm)	
	*S. aureus* TISTR 517	MRSA Isolate 2468
50 µg/mL of vancomycin	18.78 ± 0.61 *	20.09 ± 0.29 *
75 µg/mL of vancomycin	19.54 ± 0.52	21.19 ± 0.76 *
100 µg/mL of vancomycin	20.27 ± 0.41	21.98 ± 0.57
125 µg/mL of vancomycin	21.09 ± 0.41 *	22.71 ± 0.61 *
150 µg/mL of vancomycin	21.32 ± 0.36 *	23.36 ± 0.21 *
15 µg/mL of P1	19.88 ± 0.16	21.98 ± 0.35

* Significance according to Student’s *t*-test at *p*-value <0.05 compared to P1.

**Table 6:** Stability studies of anti-MRSA peptide (P1) when compared to vancomycin in various treatments.

Conditions		% Remaining Activity		
	*S. aureus* TISTR 517		MRSA Isolate 2468	
	P1	Vancomycin	P1	Vancomycin
Untreated Sample	100.00 ± 3.48	100.00 ± 4.74	100.00 ± 3.25	100.00 ± 3.24
**Thermal Stability**				
Sample 60 °C, 1 h	100.49 ± 0.84	99.25 ± 0.65	100.00 ± 0.79	98.30 ± 1.18
Sample 80 °C, 1 h	100.00 ± 0.84	97.39 ± 1.12 *	99.08 ± 0.00	98.30 ± 1.56
Sample 100 °C, 1 h	100.00 ± 0.84	94.40 ± 1.29 *	98.62 ± 0.79	96.94 ± 2.04
Sample 121 °C, 15 psi, 15 min (autoclave)	98.54 ± 1.69	91.79 ± 1.12 *	98.62 ± 0.79	93.54 ± 1.56 *
**Enzyme Stability**				
Sample + Proteinase K	94.20 ± 1.45 *	100.99 ± 2.50	91.20 ± 0.80 *	99.69 ± 1.95
Sample + Trypsin	94.20 ± 1.45 *	101.32 ± 2.63	91.20 ± 0.80 *	100.94 ± 2.17
Sample + α-Chymotrypsin	94.69 ± 1.67 *	100.33 ± 0.99	94.44 ± 0.00 *	99.06 ± 1.95
**Surfactant Stability**				
Sample + 1% SDS	114.49 ± 3.53 *	97.82 ± 1.67	117.13 ± 1.60 *	98.99 ± 0.59
Sample + 1% Triton X-100	125.23 ± 2.92 *	99.27 ± 2.18	115.74 ± 0.80 *	98.99 ± 1.17
1% SDS	114.95 ± 2.80 *	89.45 ± 2.18 *	112.96 ± 0.80 *	82.43 ± 0.59 *
1% Triton X-100	0.00 ± 0.00 *	0.00 ± 0.00 *	0.00 ± 0.00 *	0.00 ± 0.00 *
**pH Stability**				
pH 2	98.48 ± 3.17	101.74 ± 1.60	97.57 ± 1.46	100.66 ± 1.15
pH 4	97.97 ± 5.77	98.61 ± 1.21	99.03 ± 1.46	99.01 ± 0.57
pH 5	97.46 ± 2.64	97.91 ± 1.21	97.09 ± 1.68	101.32 ± 1.72
pH 6	97.97 ± 3.17	98.26 ± 2.09	97.57 ± 3.85	99.34 ± 1.99
pH 7	97.97 ± 1.76	99.65 ± 0.60	97.57 ± 5.25	100.00 ± 2.48
pH 8	102.54 ± 3.17	98.90 ± 1.90	97.09 ± 2.22	99.03 ± 0.56
pH 9	100.00 ± 1.49	99.27 ± 1.68	98.03 ± 1.71	98.05 ± 1.12
pH 11	98.01 ± 2.28	97.57 ± 3.66	98.03 ± 2.26	101.32 ± 2.05
pH 12	96.52 ± 2.28	97.09 ± 2.18	97.54 ± 0.00	96.41 ± 1.50 *
pH 13	98.51 ± 1.49	93.82 ± 2.18 *	99.51 ± 0.85	92.16 ± 0.98 *
pH 14	101.00 ± 1.72	88.00 ± 0.63 *	100.00 ± 3.08	85.29 ± 1.70 *

* Significance according to Student’s *t*-test at *p*-value <0.05 compared to the untreated samples.

**Table 7:** Sequence comparison between P1–P5 and AMPs from APD3 and DBAASP databases. The similarity was determined by MatGAT software.

Peptide	APD3 Database	Sequence	Sequence Similarity (%)	DBAASP Database	Sequence	Sequence Similarity (%)
P1	Acidocin LCHV (*Lactobacillus acidophilus* n.v. Er 317/402 Narine)	NVGVLNPPPLV	60.00	Brevilaterin V (*Brevibacillus laterosporus* S62-9)	XVXVVVKVLKYLX	53.30
P2	Bogorol L (*Brevibacillus laterosporus* MG64)	TVKIIVKVVKYLV	78.60	Brevilaterin B (*Brevibacillus laterosporus* S62-9)	XMXIVVKVLKYLX	71.40
P3	Brevibacillin (*Brevibacillus laterosporus* OSY-I1)	TLKIIVKVVKYLV	64.30	Bogorol B (*Brevibacillus laterosporus* PNG-276)	XVXIVVKVLKYLX	64.30
P4	Bogorol L (*Brevibacillus laterosporus* MG64)	TVKIIVKVVKYLV	64.30	Bogorol B (*Brevibacillus laterosporus* PNG-276)	XVXIVVKVLKYLX	64.30
P5	Brevibacillin V (*Brevibacillus laterosporus* fmb70)	TLKIVVKVVKYLV	64.30	Bogorol B (*Brevibacillus laterosporus* PNG-276)	XVXIVVKVLKYLX	64.30

## Data Availability

Not applicable.

## Supplementary Materials

The following supporting information can be downloaded at: https://www.mdpi.com/article/10.3390/molecules27238452/s1, Table S1: The b-ion and y-ion tables of each anti-MRSA peptide from *Brevibacillus* sp. SPR-20 after peptide fragmentation and mass determination by tandem mass spectrometry. (A) P1, (B) P2, (C) P3, (D) P4, and (E) P5.
