# Supplementary material for: Purification and Characterization of Novel Anti-MRSA Peptides Produced by Brevibacillus sp. SPR-20
Source: Molecules. 2022 Dec 2;27(23):8452. doi: 10.3390/molecules27238452 (PMC9738727; doi:10.3390/molecules27238452)
Supplement: Supplementary file 1 [file molecules-27-08452-s001.zip › molecules-2044414-supplementary.pdf]

Supplementary Materials: Purification and Characterization of Novel Anti-MRSA Peptides Produced by *Brevibacillus* sp. SPR-20

Nuttapon Songnaka [1], Monthon Lertcanawanichakul [2], Albert M. Hutapea [3],

Sucheewin Krobthong [4], Yodying Yingchutrakul [5], Apichart Atipairin [1, 6, *]

Table S1. The b-ion and y-ion tables of each anti-MRSA peptide from *Brevibacillus* sp. SPR-20 after peptide fragmentation and mass determination by tandem mass spectrometry. (A) P1, (B) P2, (C) P3, (D) P4, and (E) P5.

A. Peptide P1

#	b	b-H2O	b-NH3	a	c	b (2+)	Seq	y	y-H2O	y-NH3	z	z'	y (2+)	#
1	100.08	82.07	83.05	72.08	117.10	50.54	V							15
2	199.18	181.09	182.12	171.15	216.17	100.07	V	1471.96	1453.95	1454.93	1454.93	1455.94	736.48	14
3	298.21	280.16	281.19	270.22	315.24	149.61	V	1372.93	1354.93	1355.93	1355.93	1356.87	686.95	13
4	412.32	394.25	395.23	384.26	429.28	206.63	N	1273.87	1255.87	1256.87	1256.87	1257.85	637.41	12
5	511.38	493.31	494.30	483.33	528.35	256.16	V	1159.79	1141.79	1142.78	1142.78	1143.80	580.39	11
6	624.41	606.40	607.38	596.41	641.44	312.22	L	1060.73	1042.71	1043.72	1043.72	1044.69	530.86	10
7	723.48	705.47	706.45	695.48	740.50	362.24	V	947.65	929.63	930.64	930.64	931.62	474.31	9
8	851.56	833.56	834.54	823.58	868.60	426.30	K	848.58	830.57	831.56	831.56	832.54	425.30	8
9	950.63	932.61	933.61	922.65	967.67	475.82	V	720.49	702.48	703.44	703.44	704.45	360.73	7
10	1063.71	1045.69	1046.71	1035.72	1080.75	532.30	L	621.42	603.41	604.37	604.37	605.38	311.20	6
11	1160.80	1142.78	1143.80	1132.78	1177.80	580.89	P	508.34	490.33	491.29	491.29	492.31	254.66	5
12	1257.85	1239.82	1240.80	1229.83	1274.86	629.41	P	411.25	393.24	394.23	394.23	395.24	206.13	4
13	1354.93	1336.85	1337.86	1326.89	1371.91	677.94	P	314.22	296.19	297.17	297.17	298.21	157.60	3
14	1453.95	1435.94	1436.92	1425.96	1470.98	727.47	V	217.19	199.18	200.13	200.13	201.14	109.08	2
15							V	118.12	100.08	101.06	101.06	102.07	59.54	1

B. Peptide P2

#	b	b-H2O	b-NH3	a	c	b (2+)	Seq	y	y-H2O	y-NH3	z	z'	y (2+)	#
1	100.08	82.07	83.05	72.08	117.10	50.54	V							14
2	199.18	181.10	182.12	171.15	216.17	100.07	V	1517.95	1499.94	1500.92	1500.92	1501.93	759.46	13
3	330.19	312.13	313.25	302.33	347.21	165.59	M	1418.93	1400.85	1401.86	1401.86	1402.86	710.47	12
4	444.23	426.22	427.20	416.23	461.26	222.61	N	1287.89	1269.87	1270.87	1270.87	1271.82	644.42	11
5	557.31	539.30	540.29	529.32	574.34	279.16	L	1173.81	1155.80	1156.80	1156.80	1157.78	587.91	10
6	670.40	652.39	653.37	642.40	687.42	335.70	L	1060.73	1042.72	1043.72	1043.72	1044.69	530.86	9
7	769.46	751.45	752.44	741.47	786.49	385.23	V	947.65	929.64	930.60	930.60	931.61	474.31	8
8	897.56	879.55	880.53	869.56	914.59	449.28	K	848.58	830.57	831.57	831.57	832.54	425.21	7
9	996.62	978.60	979.60	968.62	1013.66	498.81	V	720.49	702.48	703.44	703.44	704.45	360.73	6
10	1109.70	1091.67	1092.69	1081.72	1126.74	555.36	L	621.42	603.42	604.37	604.37	605.38	311.15	5
11	1237.79	1219.80	1220.78	1209.81	1254.83	619.40	K	508.34	490.33	491.29	491.29	492.29	254.66	4
12	1400.85	1382.83	1383.84	1372.88	1417.90	700.43	Y	380.25	362.24	363.19	363.19	364.20	190.61	3
13	1499.94	1481.93	1482.91	1471.94	1516.97	750.47	V	217.19	199.18	200.13	200.13	201.10	109.08	2
14							V	118.09	100.08	101.06	101.06	102.07	59.54	1

C. Peptide P3

#	b	b-H2O	b-NH3	a	c	b (2+)	Seq	y	y-H2O	y-NH3	z	z'	y (2+)	#
1	100.08	82.07	83.05	72.08	117.69	50.54	V							14
2	199.18	180.79	182.12	171.15	216.17	100.07	V	1486.97	1468.96	1469.94	1469.94	1470.95	743.99	13
3	312.19	294.17	295.18	284.23	329.26	156.61	L	1387.94	1369.94	1370.88	1370.88	1371.88	694.45	12
4	426.27	408.26	409.24	398.28	443.30	214.15	N	1274.86	1256.84	1257.85	1257.85	1258.80	637.91	11
5	539.35	521.34	522.33	511.36	556.38	270.17	L	1160.79	1142.78	1143.75	1143.75	1144.77	580.40	10
6	652.44	634.43	635.41	624.44	669.47	326.23	L	1047.71	1029.70	1030.67	1030.67	1031.67	524.32	9
7	751.51	733.50	734.48	723.51	768.54	376.21	V	934.63	916.62	917.58	917.58	918.59	468.31	8
8	879.59	861.59	862.58	851.61	896.63	440.31	K	835.56	817.55	818.51	818.51	819.52	418.27	7
9	978.66	960.66	961.64	950.66	995.70	489.84	V	707.47	689.43	690.42	690.42	691.43	354.22	6
10	1091.73	1073.75	1074.73	1063.76	1108.78	546.38	L	608.41	590.37	591.35	591.35	592.36	304.69	5
11	1219.82	1201.84	1202.82	1191.86	1236.88	610.43	K	495.32	477.28	478.27	478.27	479.27	248.15	4
12	1382.89	1364.87	1365.89	1354.92	1399.94	691.96	Y	367.23	349.22	350.17	350.17	351.23	184.15	3
13	1439.94	1421.92	1422.91	1411.94	1456.96	720.48	G	204.17	186.16	187.11	187.11	188.12	102.57	2
14							K	147.11	129.10	130.10	130.10	131.11	74.06	1

D. Peptide P4

#	b	b-H2O	b-NH3	a	c	b (2+)	Seq	y	y-H2O	y-NH3	z	z'	y (2+)	#
1	100.08	82.07	83.05	72.08	117.10	50.54	V							14
2	199.18	181.10	182.12	171.15	216.17	100.07	V	1486.97	1468.96	1469.94	1469.94	1470.95	743.99	13
3	298.18	280.16	281.22	270.18	315.24	149.61	V	1387.95	1369.96	1370.88	1370.88	1371.88	694.45	12
4	412.25	394.25	395.23	384.26	429.28	206.63	N	1288.89	1270.88	1271.87	1271.87	1272.82	644.45	11
5	525.34	507.33	508.34	497.35	542.37	263.17	L	1174.81	1156.80	1157.77	1157.77	1158.79	587.41	10
6	638.42	620.41	621.42	610.43	655.45	320.15	L	1061.73	1043.72	1044.68	1044.68	1045.69	531.37	9
7	737.49	719.48	720.49	709.50	754.52	369.24	V	948.65	930.64	931.60	931.60	932.60	474.33	8
8	865.57	847.57	848.58	837.59	882.61	433.23	K	849.58	831.57	832.53	832.53	833.54	425.28	7
9	964.64	946.63	947.65	936.66	981.68	482.32	V	721.49	703.48	704.43	704.43	705.44	361.23	6
10	1077.72	1059.73	1060.73	1049.74	1094.77	539.38	L	622.42	604.42	605.37	605.37	606.37	311.70	5
11	1205.81	1187.82	1188.81	1177.84	1222.86	603.42	K	509.34	491.30	492.28	492.28	493.29	255.15	4
12	1368.87	1350.87	1351.87	1340.90	1385.93	684.94	Y	381.25	363.24	364.19	364.19	365.19	191.11	3
13	1439.94	1421.92	1422.91	1411.94	1456.96	720.49	A	218.19	200.18	201.12	201.12	202.13	109.57	2
14							K	147.11	129.10	130.10	130.10	131.09	74.06	1

E. Peptide P5

#	b	b-H2O	b-NH3	a	c	b (2+)	Seq	y	y-H2O	y-NH3	z	z'	y (2+)	#
1	100.08	82.07	83.05	72.08	117.10	50.54	V							14
2	199.18	181.10	182.12	171.15	216.17	100.07	V	1500.99	1482.98	1483.96	1483.96	1484.97	750.99	13
3	312.19	294.17	295.20	284.23	329.26	156.61	L	1401.96	1383.96	1384.89	1384.89	1385.90	701.46	12
4	426.27	408.26	409.24	398.28	443.30	213.15	N	1288.88	1270.87	1271.81	1271.81	1272.82	644.92	11
5	539.35	521.34	522.33	511.36	556.38	270.17	L	1174.80	1156.80	1157.77	1157.77	1158.77	587.40	10
6	638.41	620.41	621.42	610.43	655.45	320.15	V	1061.72	1043.72	1044.68	1044.68	1045.69	531.35	9
7	737.49	719.48	720.48	709.50	754.52	369.24	V	962.66	944.64	945.61	945.61	946.62	482.32	8
8	865.57	847.58	848.57	837.59	882.61	433.23	K	863.59	845.54	846.54	846.54	847.55	432.29	7
9	978.65	960.66	961.66	950.68	995.70	490.33	L	735.50	717.47	718.45	718.45	719.46	368.24	6
10	1091.73	1073.75	1074.73	1063.76	1108.78	546.38	L	622.42	604.38	605.37	605.37	606.37	311.19	5
11	1219.82	1201.84	1202.82	1191.86	1236.88	610.43	K	509.34	491.33	492.28	492.28	493.29	255.15	4
12	1382.88	1364.87	1365.89	1354.92	1399.94	691.94	Y	381.25	363.24	364.19	364.19	365.19	191.11	3
13	1453.95	1435.94	1436.92	1425.96	1470.98	727.47	A	218.19	200.18	201.12	201.12	202.13	109.57	2
14							K	147.15	129.10	130.10	130.10	131.11	74.06	1
